# Tetra­kis(2,4,6-tri­methyl­anilido)tin(IV)

**DOI:** 10.1107/S2414314624004796

**Published:** 2024-05-31

**Authors:** Christian Lämmer, Kurt Merzweiler

**Affiliations:** a Martin-Luther-Universität Halle, Naturwissenschaftliche Fakultät II, Institut für Chemie, Germany; Vienna University of Technology, Austria

**Keywords:** crystal structure, tin, amide

## Abstract

Transamination of Sn(NMe_2_)_4_ with H_2_NMes (Mes is 2,4,6-tri­methyl­phenyl, C_9_H_11_) led to the formation of the title compound, [Sn(C_9_H_12_N)_4_] or Sn(NHMes)_4_, which crystallizes in the tetra­gonal space group *P*  2_1_ *c*, with four formula units per unit cell. The mol­ecular structure consists of a central tin(IV) atom, which is surrounded by four NHMes groups. Sn(NHMes)_4_ possesses crystallographically imposed  symmetry. The SnN_4_ coordination polyhedron is best described as a compressed bis­phenoid.

## Abstract 2

The mol­ecular structure of Sn(NHMes)_4_ is defined by a central tin(IV) atom that is coordinated by four NHMes groups in a distorted tetra­hedral arrangement.

## Graphical abstract

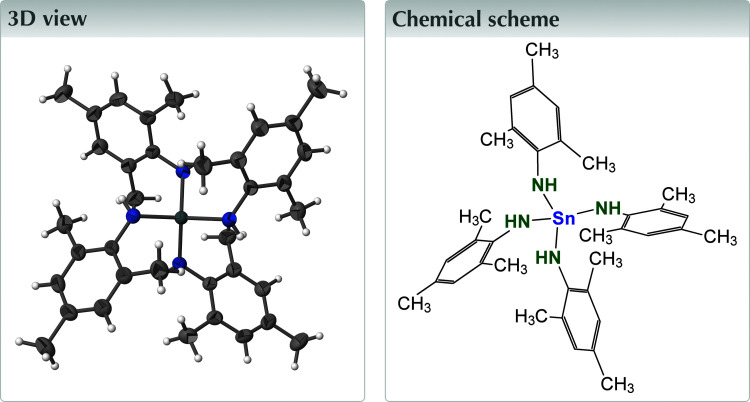

## Structure description

**Figure 1:**
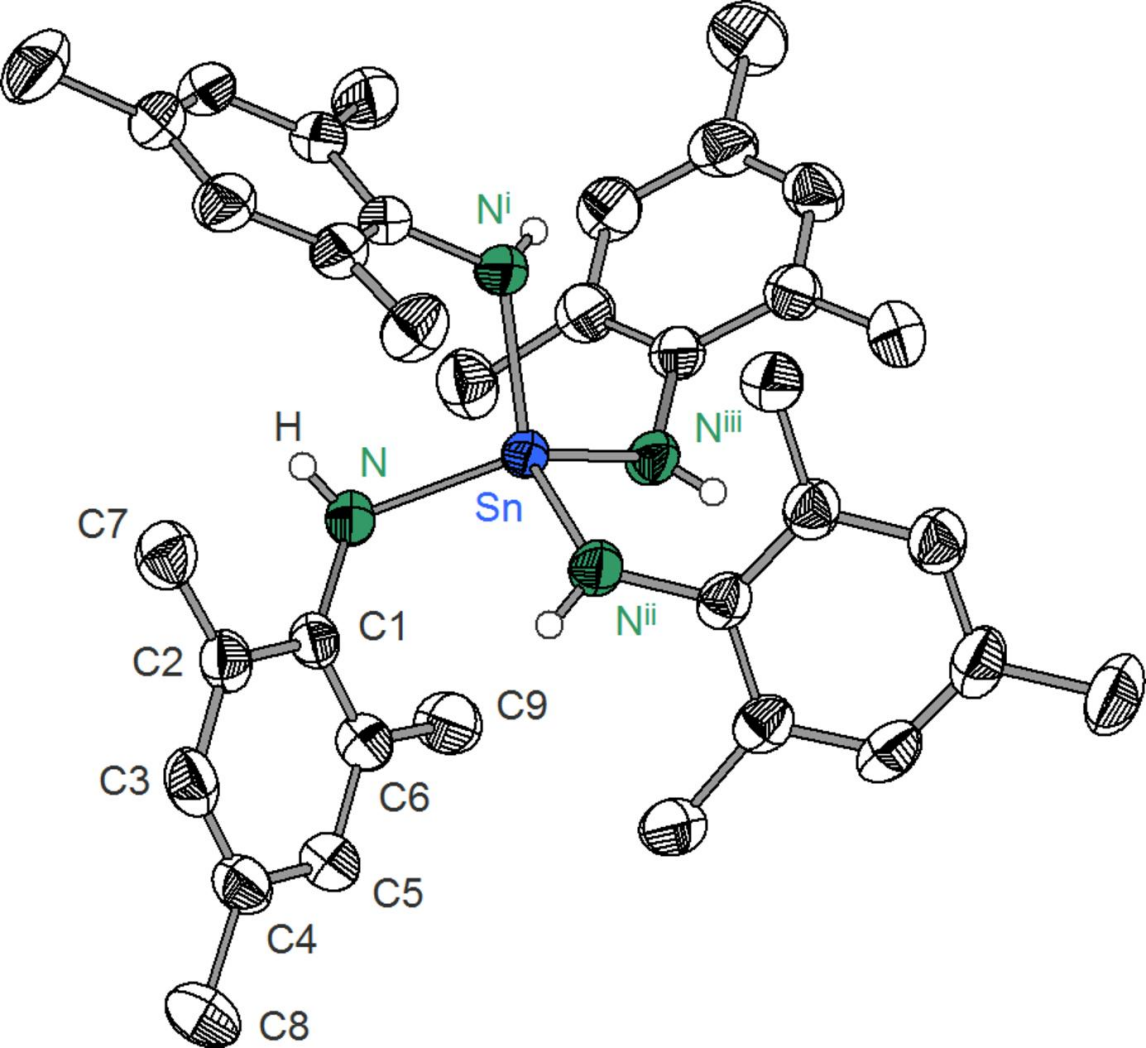
Mol­ecular structure of compound (**1**) in the crystal. Displacement ellipsoids are drawn at the 50% probability level. H atoms except for the NH group are omitted for clarity. [Symmetry codes: (i) *y*, −*x* + 1, −*z* + 1; (ii) −*x* + 1, −*y* + 1, *z*; (iii) −*y* + 1, *x*, −*z* + 1.]

Contrary to homoleptic silicon amides Si(NH*R*)_4_ [*e.g. R* = methyl (Andersch & Jansen, 1990 ▸), *R* = penta­fluoro­phenyl (Jansen *et al.*, 1992 ▸), *R* = *i*-propyl (Engering *et al.*, 2003 ▸)], corresponding tin(IV) compounds have been studied much less. In 1998, Beswick and co-workers reported the crystal structure of [Li_2_(THF)_2_Sn(NHCy)_6_] (Cy = cyclo­hex­yl), which represents a rare example of a homoleptic tin(IV) amide (Beswick *et al.*, 1998 ▸). In the context of our investigations on polynuclear organotin(IV) nitro­gen compounds like [(MeSn)_4_(NHPh)_4_(NPh)_4_] (Lämmer & Merzweiler, 1999 ▸), we found that Sn(NMe_2_)_4_ reacts with 2,4,6-tri­methyl­phenyl amine (H_2_NMes) to give the title compound, (**1**) (Fig. 1 ▸).

The crystal structure of (**1**) consists of discrete Sn(NHMes)_4_ mol­ecules without any unusually short inter­molecular contacts. The asymmetric unit consists of one tin(IV) atom on Wyckoff position 2*a* of space group *P* 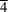 2_1_ *c* with site symmetry 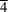 , and one NHMes unit on a general position. The tin(IV) atom exhibits a distorted tetra­hedral (bis­phen­oidal) coordination (*τ*_4_ = 0.83, with extreme values of 1 for ideal tetra­hedral and 0 for ideal square-planar coordination; Yang *et al.*, 2007 ▸) from four nitro­gen atoms with Sn—N distances of 2.033 (2) Å and N—Sn—N angles from 104.22 (5) to 120.6 (1)° (Table 1 ▸). Similar Sn—N distances were observed in (Me_3_Si)_3_CSn(NH^*t*^Bu)_3_ (2.017–2.028 Å; Janssen *et al.*, 2003 ▸), (^*t*^Bu_2_Sn)_3_(NH)_3_ (2.030 Å; Puff *et al.*, 1989 ▸) and 2,4,6-^*t*^Bu_3_-C_6_H_2_-NHSnMe_3_ (2.050 Å; Lichtscheidl *et al.*, 2015 ▸) that also exhibit four-coordinate tin(IV) atoms. In the case of [Li_2_(THF)_2_Sn(NHCy)_6_], which contains tin(IV) in a distorted octa­hedral coordination, the Sn—N distances are longer in average and vary from 2.06–2.27 Å (Beswick *et al.*, 1998 ▸). Regarding the NHMes group, bond lengths and angles are within the expected ranges. The N atom in (**1**) displays a slightly pyramidal coordination, as indicated by the sum of bond angles (345.1°).

**Figure 2:**
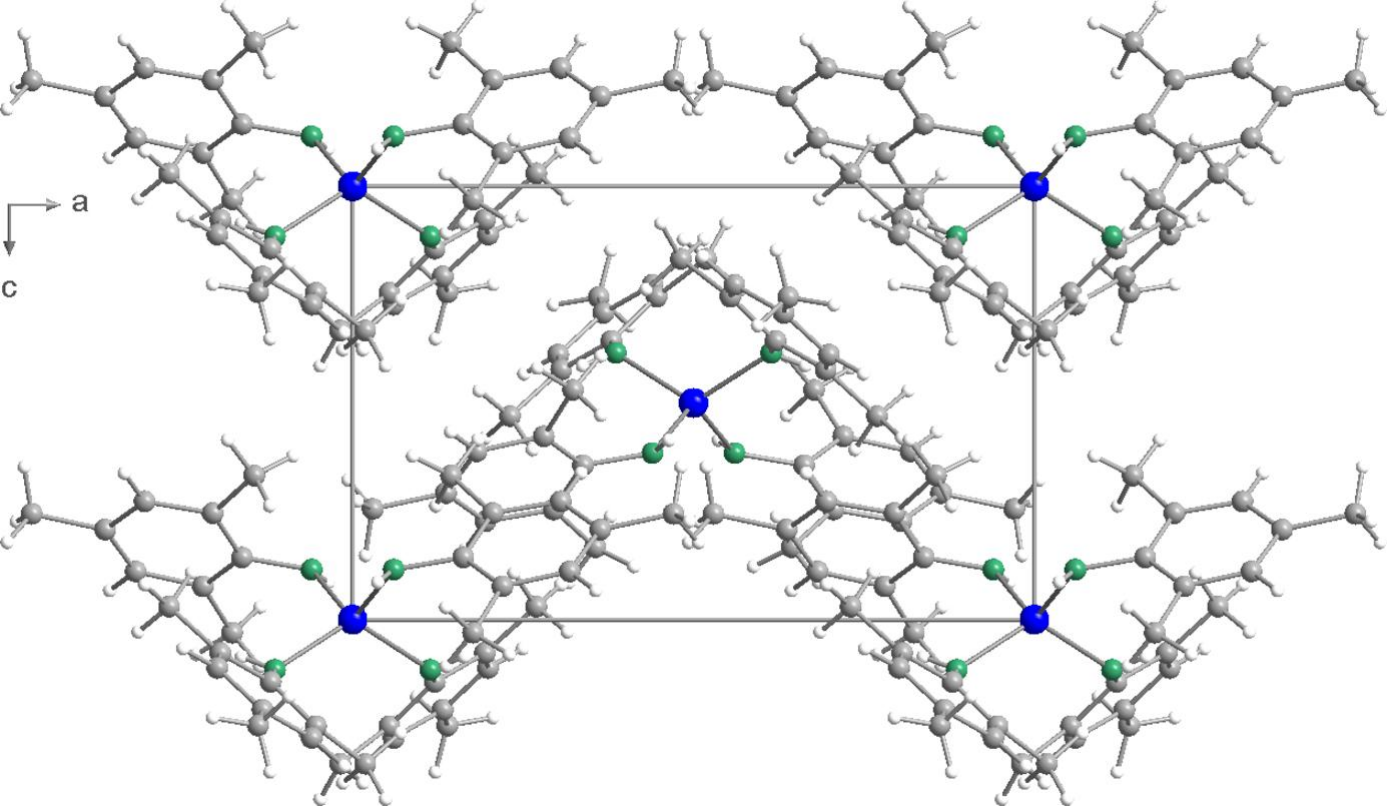
Crystal structure of compound (**1**), in a view along [010].

The packing diagram (Fig. 2 ▸) indicates that the mol­ecules of (**1**) are arranged in undulating layers parallel to (001) in the solid state. The NH groups do not participate in hydrogen bridges. This is obviously due to the steric shielding of the bulky mesityl residues.

## Synthesis and crystallization

All manipulations were carried out under an argon atmos­phere. *n*-Hexane was freshly distilled from lithium aluminium hydride. Sn(NMe_2_)_4_ was prepared according to the literature (Jones & Lappert, 1965 ▸).

2.5 g (18.4 mmol) of mesityl amine were added to a solution of 1.36 g (4.61 mmol) of Sn(NMe_2_)_4_ in 30 ml of *n*-hexane. The reaction slowly turned pale yellow and a colourless precipitate was formed. After 12 h the reaction mixture was filtered and 300 mg of the product were received. The filtrate was stored at 253 K to give another 0.83 g of yellowish crystals of the title compound. Combined yield: 1.13 g (38%).

^1^H NMR (C_6_D_6_) δ = 6.17 (*s*, C_6_ *H_2_*), 3.38 (*s*, N*H*), 2.15 (*s*, *p*-C*H_3_*), 2.07 (*s*, *o*-C*H_3_*) p.p.m. ^13^C NMR (CDCl_3_) δ = 142.1, 129.4, 128.7, 127.6, 20.4, 18.6 p.p.m. ^119^Sn NMR (CDCl_3_) δ = −170.5 p.p.m.

## Refinement

Crystal data, data collection, and structure refinement details are given in Table 2 ▸.

## Supplementary Material

Crystal structure: contains datablock(s) I. DOI: 10.1107/S2414314624004796/wm4214sup1.cif

Structure factors: contains datablock(s) I. DOI: 10.1107/S2414314624004796/wm4214Isup2.hkl

CCDC reference: 2357316

Additional supporting information:  crystallographic information; 3D view; checkCIF report

## Figures and Tables

**Table 1:** Selected geometric parameters (Å, °)

Sn—N	2.0332 (19)	N—H	0.79 (3)
N—C1	1.422 (3)		
			
N—Sn—N^i^	104.22 (5)	C1—N—Sn	122.05 (15)
N—Sn—N^ii^	120.57 (12)	C1—N—H	114 (2)

Symmetry codes: (i) 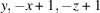 ; (ii) 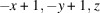 .

**Table 2:** Experimental details

Crystal data	
Chemical formula	[Sn(C_9_H_12_N)_4_]
*M*_r_	655.47
Crystal system, space group	Tetragonal, *P* 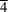 2_1_ *c*
Temperature (K)	170
*a*, *c* (Å)	13.7000 (6), 8.7123 (5)
*V* (Å^3^)	1635.21 (17)
*Z*	2
Radiation type	Mo *K*α
μ (mm^−1^)	0.81
Crystal size (mm)	0.50 × 0.35 × 0.30

Data collection	
Diffractometer	Stoe *IPDS* 2
Absorption correction	Integration [*X-RED32* (Stoe & Cie, 2016 ▸), by Gaussian integration analogous to Coppens (1970 ▸)]
*T*_min_, *T*_max_	0.735, 0.898
No. of measured, independent and observed [*I* > 2σ(*I*)] reflections	30518, 2205, 2049
*R*_int_	0.053
(sin θ/λ)_max_ (Å^−1^)	0.686

Refinement	
*R*[*F* ^2^ > 2σ(*F* ^2^)], *wR*(*F* ^2^), *S*	0.022, 0.057, 1.07
No. of reflections	2205
No. of parameters	100
H-atom treatment	H atoms treated by a mixture of independent and constrained refinement
Δρ_max_, Δρ_min_ (e Å^−3^)	0.42, −0.21
Absolute structure	Flack *x* determined using 832 quotients [(*I* ^+^)−(*I* ^−^)]/[(*I* ^+^)+(*I* ^−^)] (Parsons *et al.*, 2013 ▸)
Absolute structure parameter	−0.036 (19)

Computer programs: *X-AREA* (Stoe & Cie, 2016 ▸), *SHELXT* (Sheldrick, 2015*a* ▸), *SHELXL* (Sheldrick, 2015*b* ▸), *DIAMOND* (Brandenburg, 2019 ▸) and *OLEX2* (Dolomanov *et al.*, 2009 ▸).

## full crystallographic data

### Crystal data

**Table:**

[Sn(C_9_H_12_N)_4_]	*D*_x_ = 1.331 Mg m^−^^3^
*M**_r_* = 655.47	Mo *K*α radiation, λ = 0.71073 Å
Tetragonal, *P*42_1_*c*	Cell parameters from 28663 reflections
*a* = 13.7000 (6) Å	θ = 29.7–1.5°
*c* = 8.7123 (5) Å	µ = 0.81 mm^−^^1^
*V* = 1635.21 (17) Å^3^	*T* = 170 K
*Z* = 2	Block, colourless
*F*(000) = 684	0.50 × 0.35 × 0.30 mm

### Data collection

**Table:**

Stoe IPDS 2 diffractometer	2049 reflections with *I* > 2σ(*I*)
rotation scans	*R*_int_ = 0.053
Absorption correction: integration [X-Red32 (Stoe & Cie, 2016), by Gaussian integration analogous to Coppens (1970)]	θ_max_ = 29.2°, θ_min_ = 2.1°
*T*_min_ = 0.735, *T*_max_ = 0.898	*h* = −18→18
30518 measured reflections	*k* = −18→17
2205 independent reflections	*l* = −11→11

### Refinement

**Table:**

Refinement on *F*^2^	Hydrogen site location: mixed
Least-squares matrix: full	H atoms treated by a mixture of independent and constrained refinement
*R*[*F*^2^ > 2σ(*F*^2^)] = 0.022	*w* = 1/[σ^2^(*F*_o_^2^) + (0.0329*P*)^2^ + 0.2402*P*] where *P* = (*F*_o_^2^ + 2*F*_c_^2^)/3
*wR*(*F*^2^) = 0.057	(Δ/σ)_max_ < 0.001
*S* = 1.07	Δρ_max_ = 0.42 e Å^−^^3^
2205 reflections	Δρ_min_ = −0.21 e Å^−^^3^
100 parameters	Absolute structure: Flack *x* determined using 832 quotients [(*I*^+^)-(*I*^-^)]/[(*I*^+^)+(*I*^-^)] (Parsons *et al.*, 2013)
0 restraints	Absolute structure parameter: −0.036 (19)

### Special details

**Table:**

Geometry. All esds (except the esd in the dihedral angle between two l.s. planes) are estimated using the full covariance matrix. The cell esds are taken into account individually in the estimation of esds in distances, angles and torsion angles; correlations between esds in cell parameters are only used when they are defined by crystal symmetry. An approximate (isotropic) treatment of cell esds is used for estimating esds involving l.s. planes.
Refinement. The amino hydrogen atom was located from a difference-Fourier map and was refined freely.

### Fractional atomic coordinates and isotropic or equivalent isotropic displacement parameters (Å^2^)

**Table:**

	*x*	*y*	*z*	*U*_iso_*/*U*_eq_	
Sn	0.500000	0.500000	0.500000	0.03205 (9)	
N	0.38602 (14)	0.43981 (15)	0.3843 (2)	0.0398 (4)	
H	0.337 (2)	0.464 (2)	0.414 (3)	0.058 (9)*	
C9	0.51845 (19)	0.3557 (2)	0.1674 (3)	0.0523 (6)	
H9A	0.485199	0.412240	0.122563	0.078*	
H9B	0.547813	0.316490	0.085463	0.078*	
H9C	0.569620	0.378157	0.237652	0.078*	
C6	0.44600 (16)	0.29472 (16)	0.2542 (3)	0.0419 (4)	
C5	0.44012 (18)	0.19474 (18)	0.2270 (3)	0.0479 (5)	
H5	0.487138	0.165100	0.161838	0.057*	
C4	0.36777 (19)	0.13675 (17)	0.2921 (3)	0.0508 (6)	
C1	0.37965 (16)	0.33771 (16)	0.3564 (3)	0.0368 (4)	
C8	0.3612 (3)	0.0293 (2)	0.2557 (5)	0.0782 (10)	
H8A	0.406218	−0.007008	0.321874	0.117*	
H8B	0.378659	0.018516	0.147944	0.117*	
H8C	0.294318	0.006481	0.273615	0.117*	
C3	0.30148 (17)	0.18170 (18)	0.3885 (3)	0.0478 (5)	
H3	0.250913	0.143525	0.432859	0.057*	
C2	0.30595 (18)	0.28038 (19)	0.4231 (3)	0.0407 (5)	
C7	0.23079 (19)	0.3233 (2)	0.5301 (3)	0.0512 (6)	
H7A	0.185274	0.363947	0.471476	0.077*	
H7B	0.263505	0.363313	0.607882	0.077*	
H7C	0.194779	0.270463	0.580599	0.077*	

### Atomic displacement parameters (Å^2^)

**Table:**

	*U*^11^	*U*^22^	*U*^33^	*U*^12^	*U*^13^	*U*^23^
Sn	0.02775 (10)	0.02775 (10)	0.04063 (14)	0.000	0.000	0.000
N	0.0305 (9)	0.0343 (9)	0.0544 (11)	−0.0004 (7)	−0.0056 (8)	−0.0023 (8)
C9	0.0519 (14)	0.0485 (13)	0.0566 (13)	−0.0059 (10)	0.0118 (11)	−0.0064 (11)
C6	0.0410 (11)	0.0387 (10)	0.0461 (11)	−0.0019 (8)	−0.0044 (9)	−0.0037 (9)
C5	0.0462 (12)	0.0426 (11)	0.0548 (13)	−0.0015 (10)	−0.0049 (11)	−0.0089 (10)
C4	0.0508 (14)	0.0347 (11)	0.0668 (15)	−0.0026 (9)	−0.0158 (12)	−0.0035 (10)
C1	0.0334 (10)	0.0331 (10)	0.0440 (11)	−0.0022 (8)	−0.0081 (9)	0.0005 (8)
C8	0.074 (2)	0.0391 (13)	0.121 (3)	−0.0126 (13)	−0.007 (2)	−0.0154 (17)
C3	0.0429 (12)	0.0410 (11)	0.0596 (14)	−0.0117 (10)	−0.0119 (11)	0.0084 (11)
C2	0.0362 (11)	0.0399 (12)	0.0459 (13)	−0.0038 (9)	−0.0091 (10)	0.0047 (10)
C7	0.0431 (11)	0.0516 (13)	0.0591 (16)	−0.0067 (10)	0.0046 (10)	0.0035 (11)

### Geometric parameters (Å, º)

**Table:**

Sn—N	2.0332 (19)	C5—H5	0.9500
Sn—N^i^	2.0332 (19)	C4—C3	1.382 (4)
Sn—N^ii^	2.0332 (19)	C4—C8	1.509 (3)
Sn—N^iii^	2.0332 (19)	C1—C2	1.405 (3)
N—C1	1.422 (3)	C8—H8A	0.9800
N—H	0.79 (3)	C8—H8B	0.9800
C9—C6	1.502 (3)	C8—H8C	0.9800
C9—H9A	0.9800	C3—C2	1.386 (3)
C9—H9B	0.9800	C3—H3	0.9500
C9—H9C	0.9800	C2—C7	1.509 (4)
C6—C5	1.392 (3)	C7—H7A	0.9800
C6—C1	1.402 (3)	C7—H7B	0.9800
C5—C4	1.391 (4)	C7—H7C	0.9800
			
N—Sn—N^i^	104.22 (5)	C5—C4—C8	121.0 (3)
N—Sn—N^ii^	120.57 (12)	C6—C1—C2	119.6 (2)
N^i^—Sn—N^ii^	104.22 (5)	C6—C1—N	118.8 (2)
N—Sn—N^iii^	104.22 (5)	C2—C1—N	121.6 (2)
N^i^—Sn—N^iii^	120.57 (12)	C4—C8—H8A	109.5
N^ii^—Sn—N^iii^	104.22 (5)	C4—C8—H8B	109.5
C1—N—Sn	122.05 (15)	H8A—C8—H8B	109.5
C1—N—H	114 (2)	C4—C8—H8C	109.5
Sn—N—H	109 (2)	H8A—C8—H8C	109.5
C6—C9—H9A	109.5	H8B—C8—H8C	109.5
C6—C9—H9B	109.5	C4—C3—C2	122.5 (2)
H9A—C9—H9B	109.5	C4—C3—H3	118.7
C6—C9—H9C	109.5	C2—C3—H3	118.7
H9A—C9—H9C	109.5	C3—C2—C1	119.2 (2)
H9B—C9—H9C	109.5	C3—C2—C7	118.9 (2)
C5—C6—C1	118.9 (2)	C1—C2—C7	121.9 (2)
C5—C6—C9	120.0 (2)	C2—C7—H7A	109.5
C1—C6—C9	121.0 (2)	C2—C7—H7B	109.5
C4—C5—C6	122.3 (2)	H7A—C7—H7B	109.5
C4—C5—H5	118.9	C2—C7—H7C	109.5
C6—C5—H5	118.9	H7A—C7—H7C	109.5
C3—C4—C5	117.5 (2)	H7B—C7—H7C	109.5
C3—C4—C8	121.5 (3)		
			
C1—C6—C5—C4	3.2 (4)	Sn—N—C1—C2	114.2 (2)
C9—C6—C5—C4	−173.1 (2)	C5—C4—C3—C2	−1.0 (4)
C6—C5—C4—C3	−1.1 (4)	C8—C4—C3—C2	−179.7 (3)
C6—C5—C4—C8	177.7 (3)	C4—C3—C2—C1	0.9 (4)
C5—C6—C1—C2	−3.2 (3)	C4—C3—C2—C7	−179.7 (2)
C9—C6—C1—C2	173.1 (2)	C6—C1—C2—C3	1.2 (3)
C5—C6—C1—N	179.8 (2)	N—C1—C2—C3	178.2 (2)
C9—C6—C1—N	−3.9 (3)	C6—C1—C2—C7	−178.2 (2)
Sn—N—C1—C6	−68.8 (3)	N—C1—C2—C7	−1.2 (4)

Symmetry codes: (i) *y*, −*x*+1, −*z*+1; (ii) −*x*+1, −*y*+1, *z*; (iii) −*y*+1, *x*, −*z*+1.
